# IDENTIFYING BARRIERS TO MENTAL HEALTH SERVICES IN A RURAL COMMUNITY

**DOI:** 10.1093/geroni/igz038.2037

**Published:** 2019-11-08

**Authors:** Robert J Maiden, Danielle Gagne, Daniel l Segal, Bert Hayslip Jr.

**Affiliations:** 1 Alfred University, Alfred, New York, United States; 2 alfred University, New York, New York, United States; 3 University Of Colorado, Colorado, Colorado, United States; 4 University of North Texas, Denton, Texas, United States

## Abstract

Unmet mental health care needs of older people living in rural areas have been identified as a fundamental problem. This project engaged a rural consortium of service agencies to support recruitment through advertising, word of mouth, social media. So far, 100 rural participants aged 50 and older have completed our survey which includes the revised Barriers to Mental Health Services Scale, (BMHSS-R) which measures intrinsic barriers attributed to internal characteristics and beliefs, e.g. stigma, and extrinsic barriers, e.g. insurance costs, and lack of transportation. Preliminary results revealed increased services utilization compared to past research. However, several serious barriers remained, e.g. as lack of insurance/costs, distance/location, stigma, and lack of knowledge. The BMHSS-R results e demonstrate how the two types of barriers are related and interact within individuals. Implications are that internal barriers (e.g., stigma) and external ones (location, costs) can be reduced or eliminated through integrated medical/behavioral services.

